# Age- and gender-specific magnetic resonance imaging findings in paediatric ankle trauma with negative radiographs

**DOI:** 10.1186/s12891-025-09155-0

**Published:** 2025-09-15

**Authors:** Leyla Tümen, Gabriel Keller, Martin Brüschke, Tina Histing, Daniel Körner

**Affiliations:** 1 Department of Trauma and Reconstructive Surgery, BG Trauma Centre, Eberhard-Karls-University Tübingen, Schnarrenbergstr. 95, 72076 Tübingen, Germany; 2 Department of Diagnostic and Interventional Radiology, Eberhard- Karls-University Tübingen, Hoppe-Seyler-Str. 3, 72076 Tübingen, Germany

**Keywords:** Ankle trauma, Sprain, MRI, Child, Adolescent, Pediatric, Paediatric, Syndesmosis, Syndesmotic injury

## Abstract

**Background:**

The objective of this study is to investigate the age- and gender-specific frequencies of different pathologies after ankle trauma with negative radiographs in children and adolescents.

**Methods:**

We retrospectively analysed the MRIs of 100 patients ≤ 18 years within 30 days after acute primary ankle trauma with negative radiographs. Patients were classified into three age groups: 0–11 years, 12–15 years, and 16–18 years.

**Results:**

MRI detected pathological findings in 96 cases (96%): Lateral collateral ligament (*n* = 62), deltoid ligament injury (*n* = 10), syndesmosis injury (*n* = 12), Salter-Harris Type 1 fracture of the distal fibula (*n* = 2), bone bruise and marrow oedema (*n* = 32), soft tissue oedema and hematoma (*n* = 13), joint effusion and hematoma (*n* = 8), fracture (*n* = 12), osteochondral lesion of the talus (*n* = 2). Among LCL injuries, 21 cases (33.9%) affected the ATFL alone, 34 cases (54.8%) involved both the ATFL and CFL, 2 cases (3.2%) affected only the CFL, and 5 cases (8.1%) involved all three ligaments (ATFL, CFL and PTFL).The percentages of lateral collateral ligament and syndesmotic injuries both increased significantly with age (0–11 years: 43%, 12–15 years: 57%, and 16–18 years: 78%, *p* = 0.019; 0–11 years: 5%, 12–15 years: 5%, and 16–18 years: 24%, *p* = 0.015). There were no significant differences between the genders for any of the injuries (*p*≥ 0.539). In 24% of cases, injuries detected by MRI resulted in a modification of the clinical management.

**Conclusions:**

There is a high rate of injuries missed by radiographs but detected by MRI, with LCL injuries being the most common. The older the patient, the more likely it is that a syndesmotic injury will be detected with an MRI.

## Introduction

Lateral ankle injuries are common in paediatric and adolescent populations, especially in sports activities [1, 2]. Due to the immature skeleton, different pathologies can result from ankle inversion trauma in children compared with adults.

Recent evidence suggests that Salter-Harris Type 1 (SH1, defined in abbreviations) fractures of the distal fibula have been overdiagnosed in the past and that, in fact, lateral ankle sprains, especially affecting the anterior talofibular ligament (ATFL, defined in abbreviations), and osteochondral avulsions of the tip of the distal fibula are more frequent in the paediatric population than expected [2, 3]. The authors of a recent systematic review detected high heterogeneity in the reported rates of the different pathologies in the existing literature on lateral ankle injuries in children and adolescents [3]. SH1 fractures were found in 0–57.5% of the cases in all series, while ATFL injury was found in 3.2–80% and osteochondral avulsion of the distal fibula in 6–28.1% [3]. The authors suggested further analysis of age-specific injury patterns.

Magnetic resonance imaging (MRI, defined in abbreviations) is the gold standard in the diagnosis of acute paediatric ankle trauma. It can confirm a diagnosis when radiography and/or ultrasound are negative or unclear [4]. The treatments and rehabilitation protocols for the different ankle pathologies are different (e.g., lateral ankle sprain vs. unstable syndesmotic injury) [5]. Therefore, clarifying the final diagnosis with an MRI may be sensible.

The objective of this study is to investigate the age- and gender-specific frequencies of different pathologies primarily after ankle trauma with negative radiographs in children and adolescents. In all cases, the diagnosis was confirmed with an MRI.

## Materials and methods

### Study population

This retrospective study reviewed all consecutive ankle MRI scans performed between January 2015 and June 2023 in patients aged ≤ 18 years at a single Level 1 trauma centre. The MRI examinations were conducted using the Siemens Magnetom Aera 1.5T device from May 2019. Prior to this, the patients underwent a scan on the Philips Intera 1.5T system. The MRI examinations were performed in accordance with a standardised ankle protocol, in compliance with the guidelines established by the European Society of Musculoskeletal Radiology (ESSR, defined in abbreviations). The protocol comprised the following:

- T1-weighted turbo spin echo (TSE, defined in abbreviations) in the coronal plane (slice thickness 3 mm),
- intermediate-weighted (IMT, defined in abbreviations) TSE with fat saturation (FS, defined in abbreviations) in the coronal plane (3 mm),
- IMT TSE with FS in the transverse (3 mm) plane,
- IMT TSE with FS in the sagittal plane (3 mm),
- T2-weighted TSE in the transverse plane (3 mm), and additional.
- T2-weighted TSE sequence in the transverse/oblique plane (3 mm).

The transverse/oblique T2-weighted sequence was acquired for the optimal assessment of syndesmosis.

The decision to perform an MRI was based on the persistent clinical suspicion of a higher-grade injury that was not visible on the X-ray images, including the presence of ongoing pain, swelling, limited range of motion, and failure to improve with initial conservative management.

The indication for the MRI scan was provided by the attending surgeon (training assistant or specialist).

The following inclusion and exclusion criteria were applied:

Inclusion criteria:

- Treatment for acute ankle injury.
- Radiographs of the ankle performed without pathological findings (interpreted by an orthopaedic surgeon).
- Time between trauma and MRI ≤ 30 days.

Exclusion criterion:

- Ankle trauma in medical history.

Initially, 188 cases were identified. The protocol excluded patients with a documented history of prior trauma to the affected ankle (*n* = 34), positive radiographic findings (*n* = 39), and MRI examinations conducted more than 30 days after the initial injury (*n* = 15). Following the application of the aforementioned criteria, it was determined that one hundred patients/ankles met the inclusion criteria and were thus included in the final analysis. No formal sample size calculation was conducted; the study cohort comprised all eligible cases within the specified timeframe.

### Data capture

The following data were captured from clinical charts and physician notes documented in the hospital’s electronic medical record system: demographics (age and gender), trauma of the affected ankle in the medical history, mechanism of injury, whether the trauma occurred during sport, type of sport, reason for MRI, and time between trauma and MRI.

In 8% of cases, the trauma mechanism could not be determined based on the available documentation and was therefore recorded as “unknown”. These cases were retained in the analysis, as all other clinical information was complete.

Pathological MRI findings were rated as lateral collateral ligament (LCL, defined in abbreviations) injury, deltoid ligament injury, syndesmotic injury, SH1 injury of the distal fibula, bone bruise/marrow oedema, soft tissue oedema/hematoma, joint effusion/hematoma, occult fracture, osteochondral lesions of the talus (OCLT, defined in abbreviations), or other. Furthermore, we determined which part of the LCL had been affected.

The interpretation of all MRI examinations was conducted by consultant radiologists specialising in musculoskeletal imaging and with considerable experience in paediatric musculoskeletal MRI. Image assessments were performed independently, with the radiologists generally blinded to the detailed injury mechanism or suspected diagnosis; only the clinical indication for MRI was available at the time of interpretation. Orthopaedic surgeons were not involved in the image evaluation process.

The classification of injuries was conducted in accordance with the established MRI criteria. Specifically, Salter-Harris type I (SH1) fractures were diagnosed on the basis of the presence of physeal widening and adjacent bone marrow oedema, in the absence of a discernible fracture line [6, 7]. The identification of syndesmotic or ligamentous injuries was facilitated by the observation of discontinuity, thickening, increased signal intensity, or wavy/irregular contour of the ligaments, as well as surrounding soft tissue oedema [8, 9]. Bone marrow oedema was defined as an ill-defined area of increased signal intensity on fluid-sensitive sequences within the bone marrow [10].

Patients with multiple injuries were classified into all applicable injury categories. Consequently, individual patients may be represented in more than one category if they sustained multiple types of injury.

### Statistical analysis

Statistical analysis was performed using the software package JMP (SAS Institute Inc., JMP, Version 12.2.0, Cary, NC, USA). The Shapiro–Wilk test was applied to screen continuous data for normality of distribution. Since all continuous data were non-normally distributed, the median and range were reported. For the remaining data, frequencies and percentages were reported.

For age-specific analysis, patients were grouped into three age groups: 0–11 years (*n* = 21), 12–15 years (*n* = 42), and 16–18 years (*n* = 37). Percentages for each injury were then calculated within each age group. Furthermore, differences in the frequencies of each injury between the age groups were tested for significance with the Chi-square test. The Chi-square test was also used to detect significant differences in the frequencies of each injury between genders. The level of significance was set at *p* = 0.05 for all statistical tests.

All statistical analyses were considered exploratory.

## Results

**Fig. 1:**
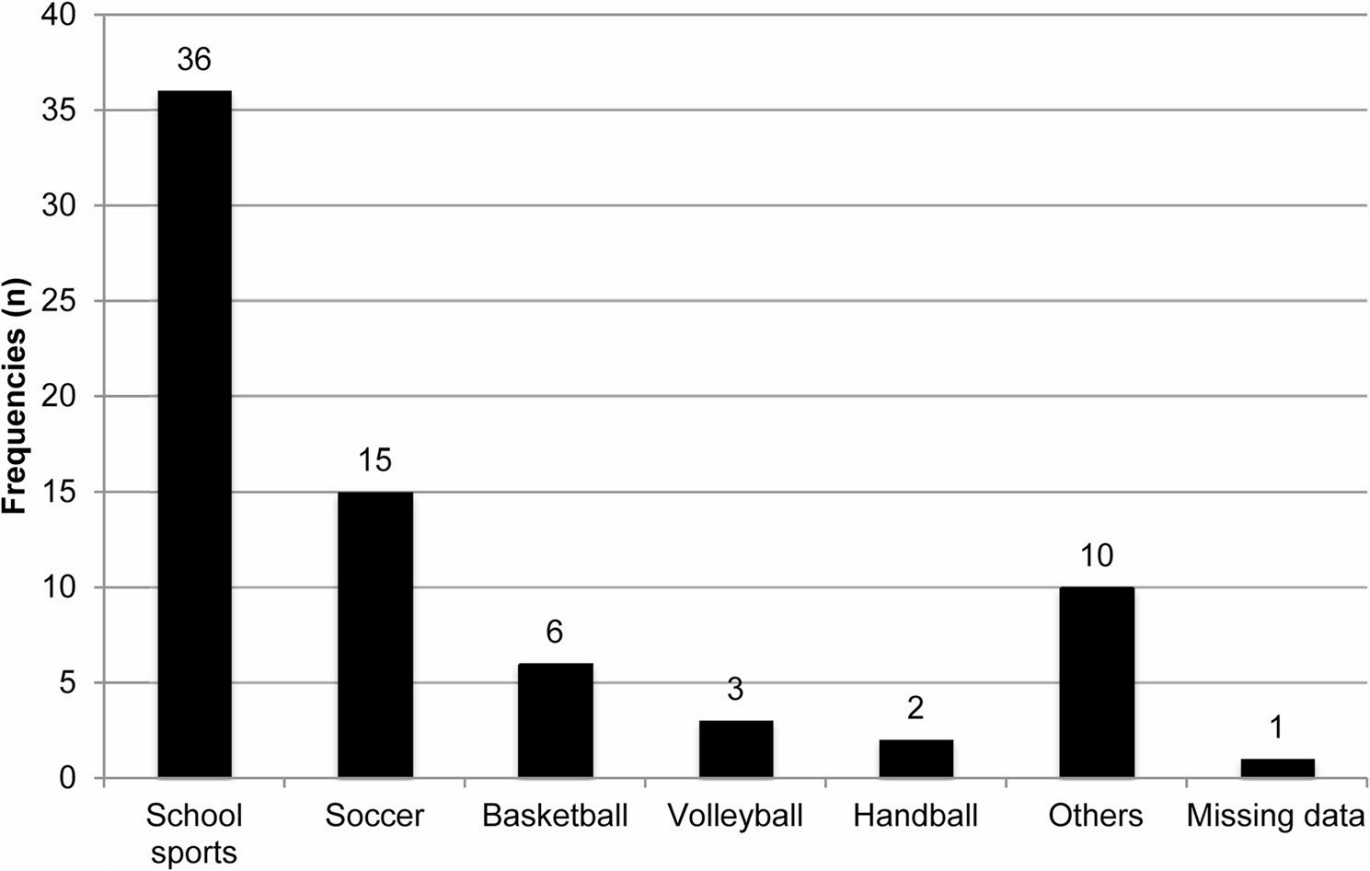
Frequencies of sporting activities (*n* = 73): Within the Group ‘Others’, each sport is represented once

The complete study population (*n* = 100) consisted of 61 boys and 39 girls (61% vs. 39%). The median age was 15 years (range 5–18 years). In 87 cases (87%), there was a twisting trauma mechanism to the ankle, and in five cases (5%), a direct blow (unknown mechanism/missing data *n* = 8). In 73 cases (73%), the trauma occurred during sports. Figure 1 illustrates the sporting activities during which the trauma occurred. In order to provide further insight into the distribution of ankle injuries across different sports, a sport-specific analysis has been included. Table 1 summarises the number of major injuries (LCL injuries, deltoid ligament injuries, syndesmosis injuries, and fractures including Salter-Harris Type 1) sustained in the most relevant sport categories (school sports, football, basketball, and volleyball). This overview demonstrates that LCL injuries were most prevalent across all sports, particularly in football and school sports, whereas other injury types were less common. Patients with multiple injuries may be represented in more than one category.

**Table 1:** Sport-Specific analysis of major ankle injury patterns

	School sports	Football	Basketball	Volleyball
LCL	22	9	4	2
Deltoid ligament	1	3	1	2
Syndesmosis	2	2	1	0
Fracture incl. SH1	4	1	0	0

This table presents the distribution of major ankle injuries by sport category among patients who sustained sports-related injuries (*n* = 73). Only major injuries and selected sport categories are shown, minor injuries and other sports are not included. Values indicate the number of patients per injury type and sport. Patients may be represented in more than one category if they sustained multiple types of injury

*LCL* lateral collateral ligament; *SH*1 Salter-Harris Type 1

All patients received radiographs of the injured ankle, which were found to be normal and devoid of any pathological findings. The reason for the MRI was suspicion of a higher-grade ankle injury, although radiographs were negative, in 94 cases (94%) and persistent complaints of the affected ankle in six cases (6%). The median time between trauma and MRI was 5.5 days (range 0–27 days).

MRI detected pathological findings in 96 cases (96%) and was normal in four cases (4%). The pathological findings are listed in Table 2.

**Table 2:** MRI findings

Injury	*N*
LCL	62
ATFL	21
ATFL + CFL	34
ATFL + CFL + PTFL	5
CFL	2
Deltoid Ligament	10
Syndesmosis	12
SH1 distal fibula	2
Bone bruise/marrow oedema	32
Soft tissue oedema/hematoma	13
Joint effusion/hematoma	8
Fracture	12
Osteochondral lesion talus	2
Others	17

*LCL* lateral collateral ligament; *ATFL* Anterior talofibular ligament; *CFL* Calcaneofibular ligament; *PTFL* posterior talofibular ligament; *SH*1 Salter-Harris type 1

The most prevalent injuries were those affecting the LCL. A total of 62 cases of LCL injury were identified, of which 21 (33.9%) involved only the ATFL, 34 (54.8%) involved both the ATFL and the calcaneofibular ligament (CFL, defined in abbreviations), 2 (3.2%) involved only the CFL, and 5 (8.1%) involved all three ligaments (i.e., ATFL, CFL, and the posterior talofibular ligament (PTFL, defined in abbreviations)) simultaneously.

The sub-analysis of the LCL injuries is also presented in Table 2.

Of the 12 cases with detected fractures on MRI, the following structures were involved: distal fibula (*n* = 3), medial malleolus (*n* = 1), talus (*n* = 1), calcaneus (*n* = 2), tarsal bones (*n* = 3), base of the fifth metatarsal (*n* = 1), metatarsals (*n* = 1), and Os trigonum (*n* = 1).

The 12 syndesmotic injuries were classified as follows: two complete ruptures, four partial ruptures, one avulsion fracture, and five cases of distension and haemorrhage. Surgical intervention was required in one case, whereas the remaining cases were managed conservatively. Specifically, one patient with a complete syndesmotic rupture underwent surgical stabilisation, whereas ten patients (including those with partial ruptures, avulsion fracture, and distension) were treated with extended immobilisation using an ankle-foot orthosis and partial weight bearing, adjusted for age and weight. For one case presenting with haemorrhage, treatment entailed the use of functional bracing coupled with full weight-bearing.

**Fig. 2:**
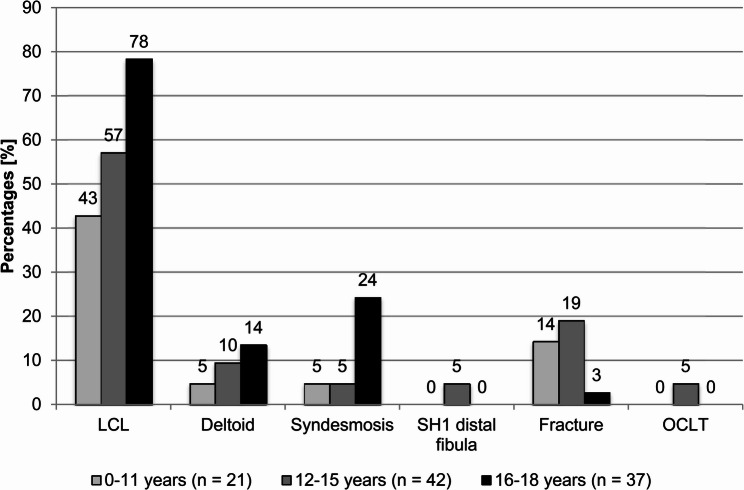
Percentages of MRI findings by age groups. LCL: 0–11 years 9/21, 34%; 12–15 years 24/42, 57%; 16–18 years 29/37, 78%; Deltoid: 0–11 years 1/21, 5%; 12–15 years 4/42, 10%; 16–18 years 5/37, 14%; Syndesmosis: 0–11 years 1/21, 5%; 12–15 years 2/42, 5%; 16–18 years 9/37, 24%; SH1 distal fibula: 0–11 years 0/21, 0%; 12–15 years 2/42, 5%; 16–18 years 0/37, 0%; Fracture: 0–11 years 3/21, 14%; 12–15 years 8/42, 19%; 16–18 years 1/37, 3%; OCLT: 0–11 years 0/21, 0%; 12–15 years 2/42, 5%; 16–18 years 0/37, 0%. LCL – lateral collateral ligament, SH1 – Salter-Harris type 1, OCLT – osteochondral lesion of the talus

In 24% of cases (occult fractures, syndesmotic and SH1 injuries), the MRI resulted in surgical or conservative treatment that went beyond ankle sprain therapy. Additionally, six of the 12 syndesmotic injuries had a concomitant LCL injury, one a concomitant deltoid ligament injury, and two concomitant LCL and deltoid ligament injuries. The two cases with SH1 injuries to the distal fibula had no concomitant injuries, and the group of ‘Other injuries’ consisted of rare, minor MRI findings. Subsequent analysis of the ‘other injuries’ revealed that 10 cases involved injuries to the capsule-ligament apparatus, including ligament ruptures, distensions and avulsions. Reactive soft tissue changes, including oedema, haematoma and muscle and tendon irritation, were identified in five cases, while cystic changes were identified in two cases. Furthermore, extensive soft tissue swelling was observed in 38 of the 96 radiographs with positive MRIs with no discernible correlation being identified. Figure 2 shows the percentages of the MRI findings ‘LCL injury’, ‘deltoid ligament injury’, ‘syndesmotic injury’, ‘SH1 injury of the distal fibula’, ‘fracture’, and ‘osteochondral lesion of the talus’ by age group. The percentages of LCL injuries were significantly different among the three age groups, with percentages increasing by age (0–11 years: 43%, 12–15 years: 57%, and 16–18 years: 78%, *p* = 0.019; absolute percentage difference 35%). This effect was also detected with deltoid ligament injuries, but the differences among the age groups were not significant (0–11 years: 5%, 12–15 years: 10%, and 16–18 years: 14%, *p* = 0.560; absolute percentage difference 9%). In addition, syndesmotic injuries were most common in the oldest age group, and the differences among the age groups were significant (0–11 years: 5%, 12–15 years: 5%, and 16–18 years: 24%, *p* = 0.015; absolute percentage difference 19%). The percentages of fractures were not significantly different among the age groups (*p* = 0.078; absolute percentage difference 5%). Both SH1 injuries of the distal fibula and OCLT only occurred in the group of 12–15-year-olds, with a low percentage of only 5% (*p* = 0.244 and *p* = 0.244, respectively; absolute percentage difference 5% in each case).

**Fig. 3:**
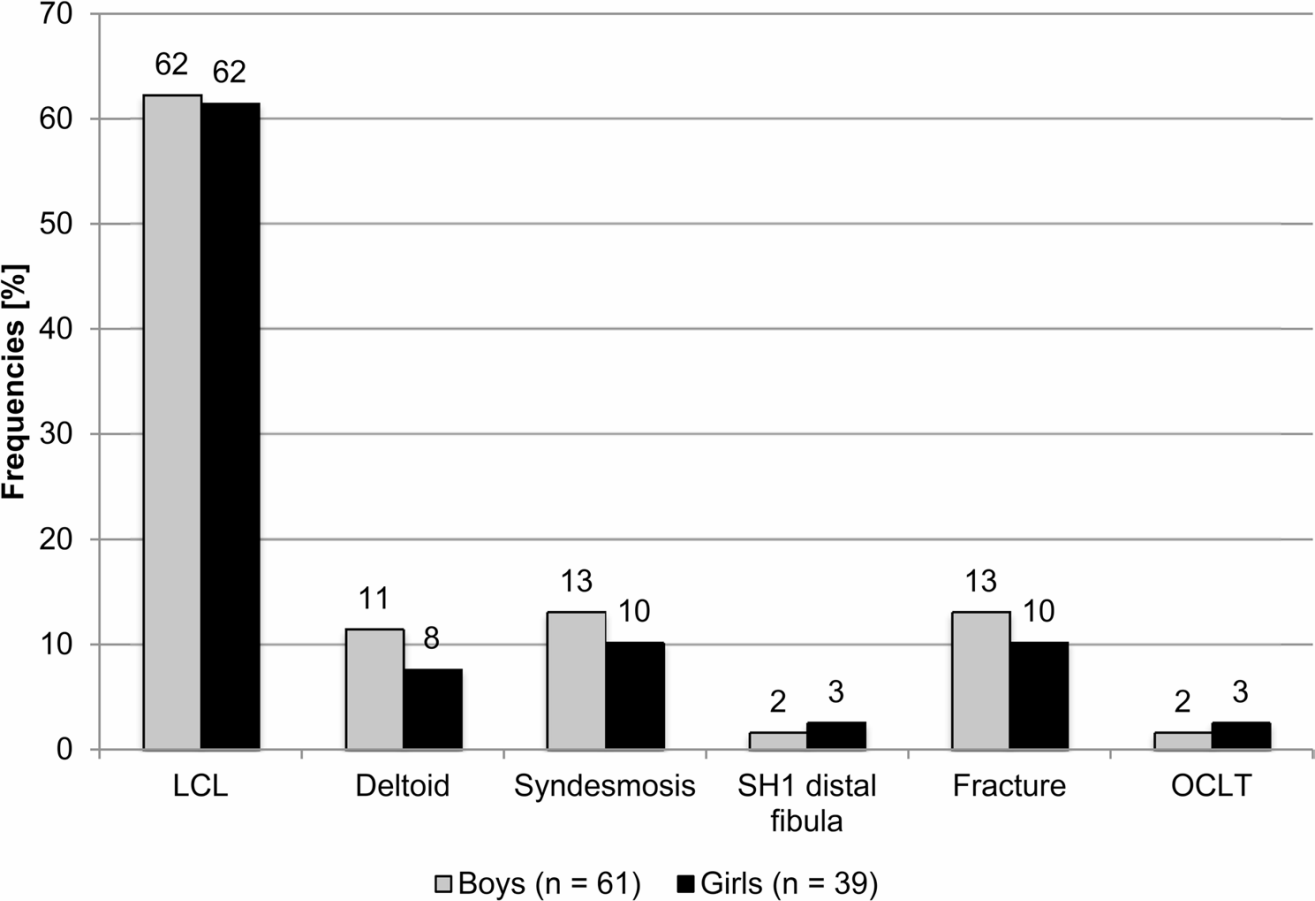
Percentages of MRI findings by gender. LCL: Boys 38/61, 62%; Girls 24/39, 62%; Deltoid: Boys 7/61, 11%; Girls 3/39, 8%; Syndesmosis: Boys 8/61, 13%; Girls 4/39, 10%; SH1 distal fibula: Boys 1/61, 2%; Girls 1/39, 3%; Fracture: Boys 8/61, 13%; Girls 4/39, 10%; OCLT: Boys 1/61, 2%; Girls 1/39, 3%. LCL – lateral collateral ligament, SH1 – Salter-Harris type 1, OCLT – osteochondral lesion of the talus

Figure 3 presents the percentages of the previously mentioned MRI findings by gender. There were no significant differences between the genders for any of the injuries (*p* ≥ 0.539).

Table 3 illustrates age-group differences in sport participation. It presents the absolute number of patients, categorised according to age group (0–11, 12–15, and 16–18 years) and sporting discipline (school sports, football, basketball, and volleyball). The data demonstrate that the highest number of injuries occurred in the 12–15-year age group, particularly in school sports, while football-related injuries were distributed across all age groups. It should be noted that this analysis does not include minor injuries or injuries sustained in other sports.

**Table 3:** Distribution of sports-Related injuries by age group and sport category

	School sports	Football	Basketball	Volleyball
0–11 years	3	5	2	0
12–15 years	20	4	1	0
16–18 years	13	6	3	3

The table presents the absolute number of patients with major sports-related injuries categorized by age groups (0–11 years, 12–15 years, 16–18 years) and selected sport categories (school sports, football, basketball, volleyball). Only major injuries and the main sports are included; minor injuries and other sports are not shown

Total number of patients per age group: 0-11 years *n* = 21; 12-15 years *n* = 42; 16-18 years *n* = 37

**Fig. 4:**
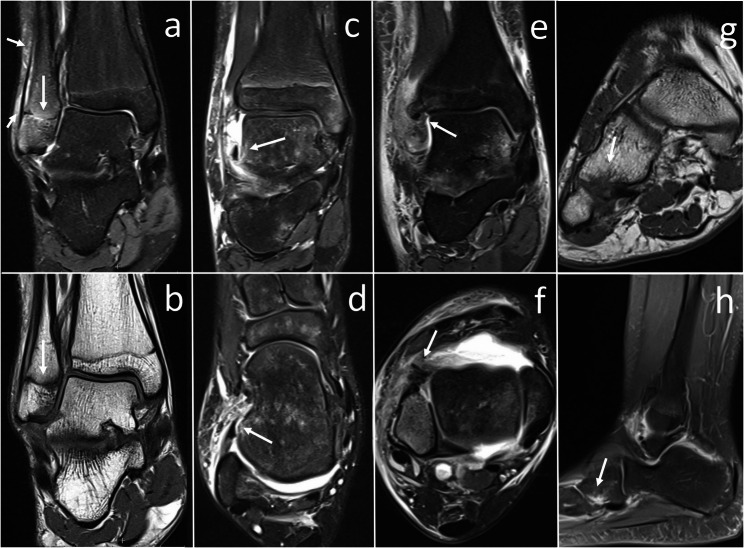
Representative MRI images of common paediatric ankle injuries. (**a**, **b**) Coronal IMT TSE images with FS and T1-weighted images in a 14-year-old female patient. Arrows indicate a SH1 fracture accompanied by an associated subperiosteal haematoma. (**c**, **d**) Coronal and transverse IMT TSE images with FS in a 10-year-old male patient. Arrows indicate a bony avulsion of the ATFL at its talar attachment, representing an injury of the LCL. (**e**, **f**) Coronal and transverse IMT TSE images with FS in a 17-year-old male patient. Arrows mark frayed and displaced fibres of the anterior syndesmosis, consistent with a syndesmotic lesion. (**g**, **h**) Coronal T1-weighted and sagittal IMT TSE images with FS in a 13-year-old female patient. Arrows highlight a bone bruise/marrow oedema and an occult impaction fracture of the cuboid. ATFL – anterior talofibular ligament; FS – fat saturation; IMT – intermediate-weighted; LCL – lateral collateral ligament; SH1 – Salter-Harris Type 1; TSE – turbo spin echo

Representative examples of the common injury patterns identified by MRI are presented in Fig. 4. These include an injury to the ATFL as an LCL injury, an avulsion of the anterior syndesmosis representing a syndesmotic injury, and infarction of the cuboid bone as an example of an occult fracture. Additionally, an SH1 injury is shown. Each pathology is depicted in two distinct imaging planes to demonstrate the characteristic radiological findings.

## Discussion

This study investigated the incidence and type of ankle injuries in 100 children and adolescents aged ≤ 18 years with negative radiographs who underwent MRI due to clinical suspicion of a higher-grade injury. MRI revealed a high prevalence of occult injuries (96%) in this cohort, with the type and incidence of injury depending on age: Ligamentous injuries were most prevalent among older children, whereas fractures predominated in younger children. Among ligamentous injuries, those affecting the LCL complex were by far the most common. Within this group, injuries to the ATFL, either isolated or in combination with the CFL, predominated, consistent with previous reports [11].

According to our study, syndesmotic injuries appear to be more prevalent among adolescents than among younger children. In 2017, Kramer et al. suggested that an open physis may offer protection against purely ligamentous syndesmotic injuries that require surgical fixation [12], which supports our results. In their study, surgical treatment of syndesmotic injuries at the ankle was most commonly associated with a closed distal tibial physis and concomitant fibula fracture, as in adults and were rare overall, accounting for only 0.96% of all paediatric ankle injuries [12, 13]. The discrepancy in incidences of syndesmotic injuries between Kramer et al.’s study and our study is caused by the different study designs, as we excluded radiographically positive cases and minor injuries not referred for MRI.

In our cohort, MRI identified a total of 28 cases of injuries to the syndesmosis, SH1 injuries of the distal fibula, fractures, or osteochondral lesions of the talus. An accurate diagnosis of such injuries is imperative for determining the most efficacious therapeutic approach, as some patients may require surgical intervention. Notably, MRI findings led to a change in management in 24% of all included cases. Specifically, among the 12 patients with syndesmotic injuries, one required surgical fixation, while ten were managed with extended immobilisation (ankle-foot orthosis) and partial weight bearing depending on age and weight. Most occult fractures and SH1 injuries resulted in cast immobilisation. Conversely, 42 patients with isolated LCL injuries were able to transition to functional bracing and early full weightbearing, indicating a de-escalation of therapy. Thus, the primary approach for radiographically negative ankle injuries is non-surgical, following a functional rehabilitation protocol. However, MRI findings may guide the necessity for prolonged immobilisation or surgical intervention in select cases [11, 14, 15].

The higher incidence of collateral ligament and syndesmotic injuries in older children, as confirmed in our study, is due to their participation in intense and competitive sports activities, resulting in greater and repetitive stresses on the ankle joint [16]. In contrast, younger children are more prone to fractures than adolescents or adults due to their stronger ligaments [17].

Aside from ligamentous injuries, avulsion fractures of the distal fibula can occur in children following ankle trauma. Contrary to the observations reported in previous studies, avulsion fractures were not identified in the radiograph-negative injuries included in this study [3, 18, 19].

We further diagnosed 12 occult fractures. However, the clinical significance of these fractures appears limited, as children with occult fractures have been reported to recover similarly to those with sprains, and management does not differ substantially [19, 20]. In a randomised, single-blind study, Boutis et al. found that a removable ankle brace was superior to a lower leg cast after 4 weeks of immobilisation in childhood ankle fractures (mainly Salter-Harris 1 and 2 injuries of the distal fibula) [21]. The feared complication of growth arrest is extremely rare in the literature, and there is no evidence that a cast can prevent such arrest [17, 21].

Similarly, bone bruising and bone marrow oedema, which occurred in 32% of our patients, did not appear to affect clinical outcomes. Our study also confirms the low rate of SH1 injuries, with just two cases to the distal fibula, in accordance with extant literature. Consequently, its impact on clinical management was negligible [3, 4, 19, 20].

The age dependency of injury patterns observed in this study is likely to reflect changes in ankle anatomy and biomechanics, as well as trauma mechanisms, during growth and development. It is hypothesised that the growth plate is weaker than bone, ligaments, and tendons as it closes during maturation [17, 22]. In order to account for variations in skeletal maturity, patients were divided into three age groups as recommended by Rougereau et al. [3]. In younger children, an open growth plate combined with relatively strong ligaments increases the risk of fractures, whereas with skeletal maturity the injury pattern shifts towards ligamentous lesions [3, 17, 23].

The onset and completion of puberty, and consequently the timing of physeal closure, vary according to gender. In girls, puberty typically commences between the ages of 8 and 13, with the peak height velocity recorded around the age of 11 and a conclusion between 14 and 16. Boys typically experience the onset of puberty between the ages of 9 and 14, reach peak height velocity around the age of 13, and the process concludes between the ages of 16 and 18 [24–26]. The susceptibility of the growth plate to injury exhibits a marked increase during periods of accelerated growth, with the peak fracture rate likely to occur concurrently with the peak height velocity [23, 27, 28]. Radiographic analysis of the distal tibia and fibula has demonstrated that complete union of the epiphyseal growth plates of these bones can occur as early as the age of 12 in female subjects, with closure observed in all female subjects by ages 15–16. In males, closure occurs slightly later, first being observed at age 13 and completed by ages 15–16 [29]. Thus, the age groups were categorised based on skeletal maturity and growth plate status, which are key determinants of injury patterns at the ankle joint [3].

Importantly, our findings indicate that radiography alone is inadequate for detecting all relevant ankle injuries in this population. Therefore, MRI may be a valuable adjunct or alternative for identifying and characterising the injuries and thereby guiding appropriate therapeutic interventions. Without MRI, more severe injuries, such as syndesmotic injuries, would be missed and treated as sprains, especially in adolescents.

However, our data also suggest that MRI may not always be necessary following ankle trauma in younger children, as it may lead to overdiagnosis and overtreatment. Therefore, the necessity of an MRI must be carefully considered, particularly since even fractures detectable only by MRI recovered similarly to sprained ankles when treated with a removable ankle brace and a self-directed return to activity [19]. With the exception of syndesmosis injuries, the treatment of ankle injuries scarcely varies.

Given these findings, the necessity of MRI in younger children must be carefully weighed to avoid overdiagnosis and overtreatment. In most cases, clinical assessment and radiography, combined with functional treatment protocols, are sufficient. While the superiority of MRI in detecting bone and soft tissue injuries is indisputable [4, 11, 15], its use should be reserved for selected cases, particularly when syndesmotic injury is suspected or initial conservative treatment fails [4, 11, 22].

The study has significant implications for preventing, diagnosing, and treating ankle injuries in that population. However, the study limitations must be considered when interpreting the results. First, a relatively small sample of 100 patients was used, which may not be representative of the entire population of children and adolescents with acute ankle injuries. To increase the statistical power and precision of the results and reduce potential bias due to sampling error, a larger sample would be necessary. Second, the gender analysis was underpowered due to uneven sample sizes, which may have limited the detection of subtle gender-specific differences in injury patterns.

Furthermore, a retrospective study was conducted based on the patients’ existing medical records, which may be incomplete or inaccurate. However, the retrospective design enabled the inclusion of a larger number of patients, which would have required a longer duration to achieve in a prospective study. Moreover, the long-term effects and cost-benefit analysis of different therapeutic and preventive interventions should be assessed.

It is important to note that our cohort included only children with clinical suspicion of complex injuries (e.g., prolonged pain, impossibility of weight bearing), rather than all ankle trauma cases. As a result, the 96% MRI abnormality rate observed in our study reflects a selected population with a higher pretest probability of occult injury and should not be extrapolated to routine ankle sprains without clinical red flags. This selection bias is a limitation of the study and may lead to an overestimation of MRI-detected abnormalities compared to the general paediatric population with ankle trauma.

## Conclusion

Despite the high prevalence of abnormalities revealed by MRI in cases of radiograph-negative ankle trauma in children, MRI results alter the management approach in the minority of cases. We do not recommend routine MRI. Conversely, MRI is advised for older children and adolescents with clinical suspicion of syndesmotic injury or persistent symptoms following ankle trauma unresolved by conservative care. This emphasises the necessity for clinical judgment in ordering MRI scans.

## Data Availability

All data generated or analysed during this study are included in this published article and are available from the corresponding author upon request.

## Abbreviations

ATFL: anterior talofibular ligament
CFL: calcaneofibular ligament
ESSR: European Society of Musculoskeletal Radiology
FS: fat saturation
IMT: intermediate-weighted
IRB: Institutional Review Board
LCL: lateral collateral ligament
MRI: magnetic resonance imaging
OCLT: osteochondral lesions of the talus
PTFL: posterior talofibular ligament
SH1: Salter-Harris Type 1
TSE: turbo spin echo
